# Supplementary material for: Alternative Lengthening of Telomeres Is Rare in Canine Histiocytic Sarcoma
Source: Cancers (Basel). 2023 Aug 22;15(17):4214. doi: 10.3390/cancers15174214 (PMC10487132; doi:10.3390/cancers15174214)
Supplement: Supplementary file 1 [file cancers-15-04214-s001.zip › Supplement Figure S1.pdf]

Suppl Figure S1

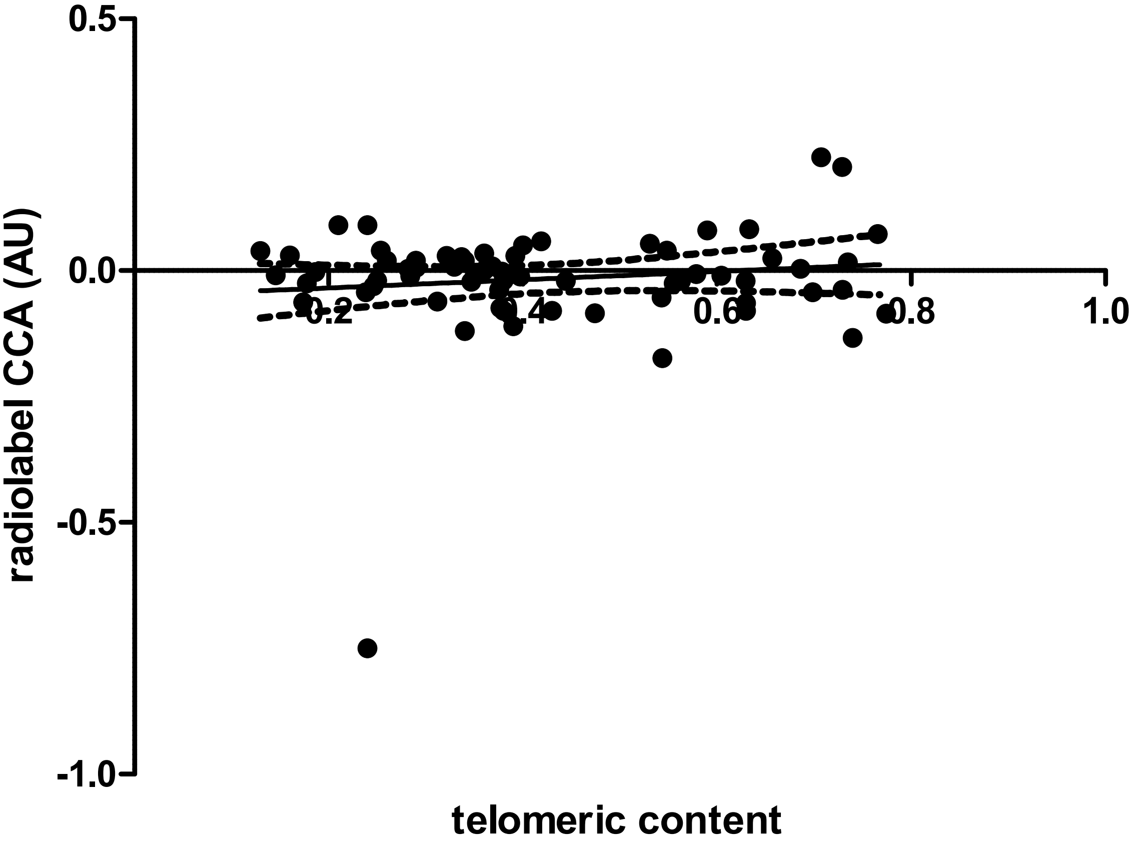

Number of XY Pairs	66
Spearman r	-0.05350
95% confidence interval	-0.2985 to 0.1981
P value (two-tailed)	0.6697
P value summary	ns
Exact or approximate P value?	Gaussian Approximation
Is the correlation significant? (alpha=0.05)	No
